# Differential Expression of the Alpha S1 Casein and Beta-Lactoglobulin Genes in Different Physiological Stages of the Adani Goats Mammary Glands

**DOI:** 10.15171/ijb.1171

**Published:** 2016-12

**Authors:** Salim Morammazi, Ali Akbar Masoudi, Rasoul Vaez Torshizi, Abbas Pakdel

**Affiliations:** ^1^Department of Animal Science, Faculty of Agriculture, Tarbiat Modares University, Tehran, Iran; ^2^Department of Animal Science, Faculty of Agricultural and Natural Resources, University of Persian Gulf, Bushehr, Iran; ^3^Department of Animal Science, Isfahan University of Technology, Isfahan, Iran

**Keywords:** Adani goats, Alpha S1 casein, Beta-lactoglobulin, Gene expression, Mammary gland, Milk yield breeding value

## Abstract

**Background:**

Milk proteins genes have been the focus of the researches as the candidate target genes that play a decisive role when animal breeding is desired.

**Objectives:**

In the present study, the transcriptional levels of Beta-lactoglobulin (*BLG*) and Alpha S1 casein (*CSN1S1*) genes were investigated during prenatal, milking and drying times in mammary glands of the Adani goats which showed high and low breeding values.

**Materials and Methods:**

The breeding values of the animals were estimated first by applying multi-trait random regression model. Using the biopsy gun, the mammary gland samples were taken and real-time PCR was applied to search the expression of the genes. Fixed factors of the model were the breeding value groups, sampling times and their interactions.

**Results:**

The interactions were significant for both genes. At milking time, the high breeding value group exhibited more transcriptional levels for *BLG* and less transcriptional levels for *CSN1S1* gene compared with the low breeding value group. The expression patterns of these genes were also different between the two breeding value groups. The maximum level of *BLG* and *CSN1S1* transcriptions were found to occur at drying time.

**Conclusions:**

A difference in the gene expression was observed between the two groups which indicate the change in the nucleotide sequence for transcription factor binding sites, or miRNA binding sites, otherwise in the coding regions. Therefore, the variations in the coding and promoter regions of this gene should be investigated in the further studies.

## 1. Background

In the past decades, detection of quantitative trait loci (QTL) and genes affecting on milk yield traits has been the focus of various molecular genetics studies (1). To date, the association between the milk yield traits and several genes have been investigated in livestock (2,3). The milk protein genes encode proteins which are produced and secreted by the mammary glands epithelial cell (4). These genes have taken an important role and were the focus of analysis as well as manipulation in animal breeding. As well, their polymorphisms were used in the analysis of the genetic diversity, phylogenetic studies, genomic selection, and conservation strategies (5). Moreover, mutations that have occurred within these genes have an effect on the level of gene transcription and milk yield traits (6).

More than 95% of the proteins in the milk of goat and other livestock are encoded by casein and whey protein genes (7). Many studies have proven that there are four casein proteins in the goat's milk. These genes are encoded by four tightly linked casein genes (*CSN1S1*, *CSN2*, *CSN1S2*, and *CSN3*) within a 250 kb of the gene cluster mapped on the chromosome 6(CHI6) (8). These genes significantly affect the physical, chemical and the nutritional quality of the goat’s milk (7). The caprine casein genes are highly polymorphic.

Among which, *CSN1S1*, encoding αs1-casein protein which is the most variable, having at least 18 variants with strong, medium, and weak effects on the protein content, as well as null alleles characterized with no protein synthesis (9,10). The most genetic variants differ only by amino acid substitutions, however, a number of variants arise from exon skipping (11,12), as well. The caprine *CSN1S1*, with an mRNA of 657 bp in length encodes 213 amino acids precursor. CSN1S1 protein is a highly phosphorylated and calcium sensitive protein with an important role in the capacity of the milk for transporting calcium phosphate (13). Sanchez *et al*. (2005) have predicted that the *CSN1S1* gene could be used as a candidate gene for improving milk protein traits (14).

The two main whey proteins are alpha-lactalbumin and beta-lactoglobulin (BLG). The BLG is the major whey protein in the milk of ruminant. BLG could also be observed in the milk of different mammalian animals including dogs, cats and dolphins. However, BLG is lacking in the human milk (15). BLG belongs to a family of lipocalins, a group of small proteins with some particularities such as the capability to bind hydrophobic molecules (16). It has been suggested a transport of retinol and fatty acids for physiological functions of BLG (16). Though, the general affinity of BLG with these hydrophobic particles did not allow attributing a particular function (17).

The sequence of *BLG* gene has been determined in the goat and mapped on CHI11 (18) and harbors seven exons along with six introns that encode a 162 amino acids protein (19). Many genetic polymorphisms in the promoter and coding region of this gene have been reported in goat (20,21), sheep (22,23), cattle (2,24), and buffalo (25). BLG and CSN1S1 are very important in the milk industry and animal breeding. Although these genes have been studied from different points of view, but no report was found in the transcriptional level of the gene in the mammary gland tissue of the goat.

## 2. Objective

The purpose of the present study was to assess the effect of breeding value groups on transcriptional levels of *CSN1S1* and *BLG* genes in diverse physiological periods in mammary gland of Adani goats.

## 3. Material and Methods

### 3.1. Animals

The animals have been raised by Adani Goat Breeding Center as described elsewhere (26). Briefly the source of animals was created by collecting the goats from different regions of Bushehr province in 2005. The goats were treated under local conditions and management and fed using natural pastures. Moreover, dairy goats were given additional food in autumn. Adani goats are polyestrous, but their major mating seasons are early spring to the early summer and end of summer to the middle of autumn. Generally the mating system of the herd is natural.

### 3.2. Genetic Assessment of Animals

Genetic evaluation of the animals was performed as described previously (26). Genetic assessment of the animals was carried out using daily milk records of the first, second and the third lactations. Briefly the data of 2364 milk test day records from 375 goats were used. We firstly run several models with different degree of Legendre polynomial function for additive genetic and permanent environmental effects and then the multitraits random regression model with the fifth-order Legendre polynomial function was selected for genetic assessment analysis of both additive genetic and permanent effects.

Using the above model, data of each lactation is different but correlated traits were assessed. Residual effects of the observations were categorized in to the 6 periods in milk (DIM) classes within parity (DIM 5 to 17, 18 to 32, 33 to 47, 48 to 62, 63 to 77, and 78 to 90).

The WOMBAT program was used to analysis the data (27). Because the main goal of the current research was the analysis of the gene expression in the first lactation, thus the breeding value of the first lactation was estimated and the following equation was used to calculate the coefficients for the first trait:

$$u_{i}=\sum_{5}^{90}\phi_{r}^{}\alpha_{ir}$$

In this formula, u_i_ is the aggregated breeding value between 5 to 90 DIM for i^th^ animal, Φr is the matrix of Legendre polynomials estimated from 5 to 90 DIM, and α_ir_ is the breeding value coefficients of ith animal in the first lactation. In order to estimate the milk yield breeding values for nulliparous goats, as used for gene expression analyses, the milk yield breeding value of their parents were employed using the following equation:

u_o_ = u_s_ + u_d_

In this equation uo, us, and ud are the aggregated milk yield breeding value for offspring and parents, respectively.

Consequently, three goats with the highest breeding values and three goats with the lowest breeding values were selected randomly for gene expression analyses.

### 3.3. Tissue Sampling, RNA Extraction and Relative Real-Time PCR

Mammary gland tissues were taken by biopsy gun at prenatal, milking and drying periods as illustrated previously (26). Samples were kept at –80°C until RNA extraction. Total RNAs were extracted from the samples with RNX plus (Cinna Clon Inc., Iran) and the cDNAs were synthesized from 2 μg of total RNA using RT-PCR kit (Vivantis, Malaysia).

To measure the gene expression of the *BLG* and *CSN1S1*, real-time PCR reaction was used (28,29). Real-time PCR reaction was carried out in a total volume of 20 μL including cDNA, 5X HOT FIREPol® EvaGreen®qPCR Mix Plus with ROX (Solis BioDyne, Estonia), related forward and reverse primers and distilled water, by using a Miniopticon real-time PCR system (Bio-Rad Laboratories, USA). Glyceraldehyde-3-phosphate dehydrogenase (*GAPDH*), was used as the reference gene (30-32). The oligonucleotide sequences of the primers for the candidate genes were as follows: *CSN1S1*: 5′-CACAGTATGAAAGAGGGAAAC, 5′-ATGGGATTAGG GATGTCAGAG; *BLG*: 5′-GACTTGGTACTCCTTGGCTAT, 5′-TTGAACACCGCAGGGATCTTG; GAPDH: 5′- AGTCAAGGCAGAGAACGGGAA, 5′-ACAAACATG GGGGCATCAGCA. Real-time PCR reactions were performed as described previously (26).

### 3.4. Statistical Analysis

The data of the real-time PCR were analyzed as described elsewhere (26). Briefly the difference (ΔCt) between the threshold cycle (Ct) for *GAPD H* and that of in the candidate gene (*CSN1S1* or *BLG*) was used to evaluation of the gene expression variations. The following statistical model was applied for the analysis of data:

y_ijk_= S_i_+B_j_+(S×B)_ij_+a(B)_k_+(S×a(B)_ik_+e_ijko_

Where, y_ijk_ is the difference (ΔCt) between the threshold cycle (Ct) for *GAPDH* and the gene of interest, S_i_ is fixed effect of the sampling times, B_j_ is fixed effect of the breeding value groups, a(B)_k_ is random effect of the individual animal within breeding value groups and (S*a(B))_ik_ is random effect of the interactions of the breeding value groups with sampling times. Data were analyzed by GLM procedure by using SAS 9.1 software (SAS Institute Inc.; Cary, NC).

## 4. Results

### 4.1. BLG Gene Expression

**Figure 1:**
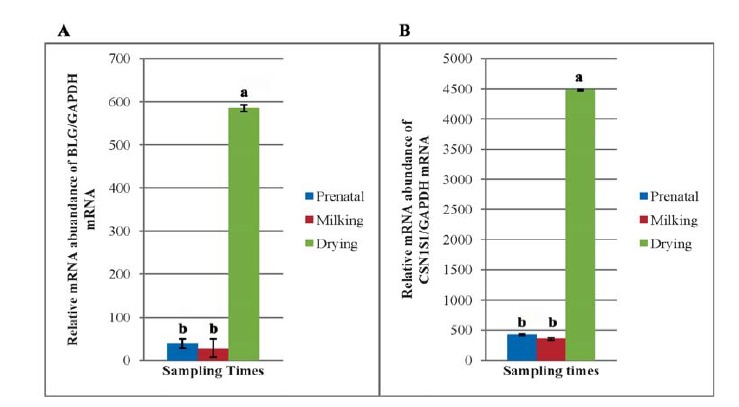
A comparison between the transcription levels of the two studied genes: A: *BLG* and B: *CSN1S1* throughout different sampling times

**Figure 2:**
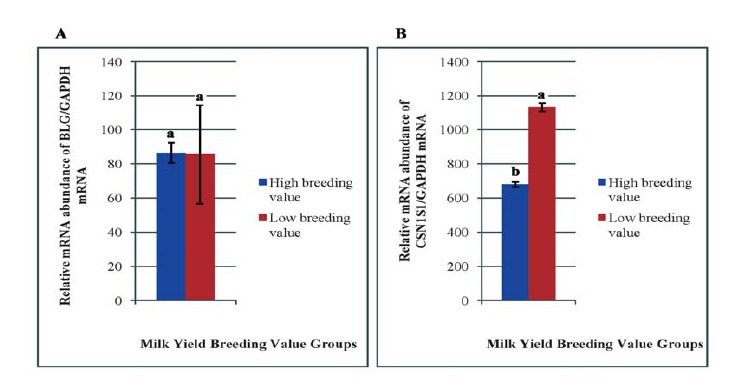
High and low milk breeding value groups and the level of A: *BLG* and B: *CSN1S1* genes’ transcription. While the overall average of BLG expression in all stages is not different between milk yield breeding value groups, the CSN1S1 is highly expressed in low milk yield breeding value groups

**Figure 3:**
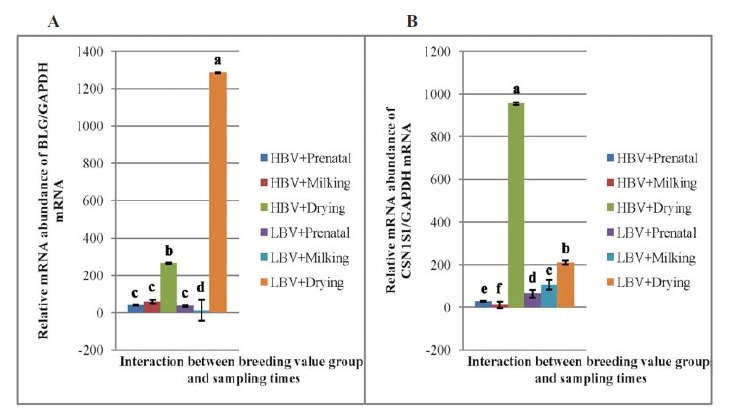
Breeding value groups and sampling stages interactions in relation with expression levels of the two studied genes: A: *BLG* and B: *CSN1S1*. HBV=high breeding value group, and LBV=low breeding value group, respectively

The result obtained for *BLG* gene expression has indicated that sampling time and the interaction with breeding value groups had a significant effect on *BLG* transcriptions. Although the expression values for *BLG* mRNA did not significantly differ between the prenatal and milking samples (Figure 1A), but mRNA abundance at drying time samples was increased significantly in comparison to those of the prenatal and milking samples (Figure1A). As shown in Figure 2A the *BLG* transcriptions did not show a difference in the expression level between the high and low breeding value groups, however, transcriptions were affected by the breeding value groups and sampling periods interactions (Figure 3A).

As could be observed in figure 3A, high and low breeding value groups exhibited a similar amount of BLG mRNA in the prenatal step (P>0.05). However, in milking time, transcriptions abundance of *BLG* in high breeding value group was more than that of low breeding value group (Figure 3A). The BLG mRNA in the high breeding value group was less than that of low breeding value group in drying time; vice versa (Figure 3A).

The gene expression patterns of *BLG* were different between high and low breeding value groups (Figure 3A). In high breeding value group, similar amounts of BLG mRNA were observed in prenatal and milking samples. In addition, the maximum expression was observed in drying time. In low breeding value group, the amount of BLG mRNA were decreased from the prenatal to the milking time and then increased in time of drying.

### 4.2. CSN1S1 Gene Expression

Analysis of *CSN1S1* gene expression and comparison between the breeding value groups in different physiological states has indicated that all factors have significantly affected the *CSN1S1* gene expression. As could be concluded from the figure 2B, the expression pattern of *CSN1S1* in high breeding value group was less than that of low breeding value group (P<0.01). In addition, sampling times has also affected the abundance of transcriptional level of the *CSN1S1* gene (P<0.01), and mRNA of *CSN1S1* gene was higher in drying period compared to the prenatal and milking periods (Figure 1B). The amount of CSN1S1 mRNA at prenatal and milking times was similar (P>0.05).

As shown in figure 3B, the abundance of CSN1S1 mRNA could be affected by the interactions between breeding value groups and sampling times (P<0.01). Significant differences were observed in CSN1S1 mRNA in prenatal, milking, and drying times between the high and the low breeding value groups (figure 3B). At the prenatal and milking time, the amount of CSN1S1 mRNA was lower in the high breeding value group compared to the low breeding value group (figure 3B). However, the abundance of *CSN1S1* transcriptions was higher in the high breeding value group during the drying time (figure 3B).

The gene expression patterns of *CSN1S1* are shown in figure 3B for both breeding value groups. In high breeding value group, the amount of CSN1S1 mRNA decreased from the prenatal to the milking times and increased in drying time compared to the prenatal and milking times. In the other group, the abundance of CSN1S1 mRNA was increased from prenatal to the milking time and drying time, as well.

## 5. Discussion

In the present study, through comparison of the breeding value groups with transcription levels in different times of Adani goats production we have shown that the amount of BLG and CSN1S1 transcription have been affected significantly by the breeding value groups; with an exception for *BLG* gene in the prenatal time. The BLG transcriptional abundances, unlike *CSN1S1* gene, did not exhibit differential expression between the high and the low breeding value during the prenatal time. This feature might take its root from the similar physiological and transcriptional conditions that have influenced the BLG transcription in the mammary gland of the both breeding value groups.

Breeding value of an animal is the sum of the additive genetic effects of the genomic loci related to the interested trait. The additive genetic effect is associated with the substitution of one genotype with another genotype within an interbreeding population. Therefore, the variations in genomic loci are associated with the high and the low breeding values. At milking and drying times, the amount of CSN1S1 and BLG transcriptions were different between the two breeding value groups. The breeding values of the animals were estimated based on milk yield. Therefore, these differences are very important as they affect *CSN1S1* and *BLG* genotypes on transcriptional level, milk yield, and proteins that have been reported (2,33-35). In addition, the differential expression between breeding value groups may be caused by genetic variation that is related to breeding value groups.

Some evidences have indicated that genetic differences in the coding region can alter the expression of genes at proteins levels (36). These variations might have been deleted the exon and have led to a truncated protein in the milk (36). In addition, they could influence the rate of synthesis of the corresponding protein (37) or silent mutations in the coding region (9). Furthermore, changes in the coding region can affect the mRNA stability; resulting in the different amount of measurable transcriptions.

The genetic variations in the upstream region of the gene could be associated with the differential expression of *BLG* and *CSN1S1* genes (24,38) too. Many variations have been reported in the promoter region of these genes in goat (21,39-41) which might be in correlation with the gene expression. Furthermore, the regular elements of the gene expression are positioned in the promoter region. Therefore, any change in the nucleotide sequence of these regions changes the transcriptional levels of these genes, accordingly. Ganai *et al*. (2009) have found 50 SNPs in the regulatory and coding region of bovine *BLG*, in which one of these SNPs affected the BLG protein concentration (24). Braunschweig and Leeb (2006) have shown that the differential expression was affected by a C to A transversion (34). These polymorphisms in the upstream regions of the *BLG* and *CSN1S1* genes can affect the binding activity of the related response elements and consequently have an impact on the expression pattern of the gene products (38,39). Furthermore, numerous potential binding sites for several transcription factors (TFs) were found within the *BLG* and *CSN1S1* promoter sequence (42,43). The genetic variation in these sites may alter expression of *BLG* and *CSN1S1* genes respectively. Sardina *et al*. (2012) have reported that the promoter region of the *BLG* gene in Sicilian goat breeds contains a high intensity of the genetic variation because of the presence of more than 35 single nucleotide polymorphisms. They indicated that a number of these SNPs are located in MPBF (milk protein binding factors), NF-I (nuclear factor-I), and AP-2 (activator protein-2) binding sites (21). Prinzenberg *et al*. (2003) have revealed that mutations in several *CSN1S1* alleles can alter the potential of transcription factor binding sites of ABF1, OCT1, AP1, and YY1 (42). Kuss *et al*. (2005) have shown that the association between bovine *CSN1S1* promoter variants and its transcription is probably to be resulted by a lower affinity of the related response element to a c-jun-containing *CSN1S1* dimer with repressor possessions (38). So, the existence of SNPs in *BLG* and *CSN1S1* promoter region could change the binding affinity of transcription factors and consequently could influence the BLG and CSN1S1 transcriptional level.

As shown in figure 3B, at milking time, the transcriptional levels of *CSN1S1* gene in low breeding value group is more than that of in high breeding value groups. This result is in agreement with the effects reported by Barbieri *et al*. (1995) (44). They have reported that the genotypes (for *CSN1S1* locus) that produced low milk yield are associated with high protein yield.

The maximum transcriptional levels of *BLG* and *CSN1S1* genes at the drying time were observed in low and high breeding value groups, respectively. This observation is in contrast to what observed at the milking time. The differences that are observed between breeding value groups at milking and drying time might be due to the large variation in the binding sites for several transcription factors that were located within the promoters structure (38,43).

Differential expression of TFs was observed at milking and drying times such as STAT5 and STAT3 (45,46). Moreover, significant differences in the expression of the 56 microRNAs was reported in the lactating mammary gland compared to non-lactating mammary glands (47).

Regarding the importance of the promoter and coding regions in the activity of the genes and the differential expression that observed between high and low breeding value groups, it could be explained that the structure of genes should be evaluated in the population of the Adani goats. Moreover, it could be said that the animal selection in Adani goats based on the milk breeding value would be affected the BLG and CSN1S1 proteins in milk. High amounts of these proteins are necessary for the milk industry and cheese yield, thus, identification of variable regions that are associated with the high expression could be helpful for this industry. On the other hand, BLG and CSN1S1 are the potential allergens for some peoples (48), therefore, milk production with few amount of these proteins might be used for people with allergies to these milk proteins. In this regard, it is necessary to detect the genotypes that are associated with the null or faint expression of these genes.

BLG protein has a role as a carrier of fatty acids and retinol, but its general affinity with these hydrophobic molecules did not permit to ascribe a BLG particular role in the mammary gland (17). Furthermore, the large BLG transcription in drying time as found in this study validates the importance of BLG in this period. Consequently, the best time to identify the BLG specific role is drying period. While different content of BLG protein in the milk of the goat was detected (49), but the synthesis of BLG has not been reported at prenatal and drying times.

## 6. Conclusions

In this study, the expressions of *BLG* and *CSN1S1* genes were significantly different between the high and the low breeding value of Adani goats at milking and drying times. In addition, different expression was observed at the considered physiological times, and the maximum of gene transcriptions was detected at drying time. Therefore, these genes are proper candidate genes and suggested to be considered in Adani goat breeding program to improve milk yield for the dairy industry and people with allergies to milk proteins.

## Acknowledgements

The authors would like to thank Adani goat breeding Center for their valuable helps in collecting the samples.

## References

[1] Sanders K, Bennewitz J, Reinsch N, Thaller G, Prinzenberg EM, Kuhn C (2006). Characterization of the DGAT1 mutations and the CSN1S1 promoter in the German Ange ln dairy cattle population. J Dairy Sci.

[2] Yang F, Li L, Liu H, Cai Y, Wang G (2012). Polymorphism in the exon 4 of beta-lactoglobulin variant B precursor gene and its association with milk traits and protein structure in Chinese Holstein. Mol Biol Rep.

[3] Yue XP, Zhang XM, Wang W, Ma RN, Deng CJ, Lan XY (2011). The CSN1S1 N and F alleles identified by PCR-SSCP and their associations with milk yield and composition in Chinese dairy goats. Mol Biol Rep.

[4] Aggeler J, Park CS, Bissel l MJ (1988). Regulation of milk protein and basement membrane gene expression: the influence of the extracellular matrix. J Dairy Sci.

[5] Rout PK, Kumar A, Mandal A, Laloe D, Singh SK, Roy R (2010). Characterization of casein gene complex and genetic diversity analysis in Indian goats. Anim Biotechnol.

[6] Hayes B, Hagesaether N, Adnoy T, Pellerud G, Berg PR, Lien S (2006). Effects on production traits of haplotypes among casein genes in Norwegian goats and evidence for a site of preferential recombination. Genetics.

[7] Martin P, Szymanowska M, Zwierzchowski L, Leroux C (2002). The impact of genetic polymorphisms on the protein composition of ruminant milks. Reprod Nutr Dev.

[8] Dagnachew BS, Thaller G, Lien S, Adnoy T (2011). Casein SNP in Norwegian goats: additive and dominance effects on milk composition and quality. Genet Sel Evol.

[9] Caroli A, Chiatti F, Chessa S, Rignanese D, Ibeagha-Awemu EM, Erhardt G (2007). Characterization of the casein gene complex in West African goats and description of a new alpha(s1)-casein polymorphism. J Dairy Sci.

[10] Swalve HH (2000). Theoretical basis and computational methods for different test-day genetic evaluation methods. J Dairy Sci.

[11] Brignon G, Mahe MF, Ribadeau-Dumas B, Mercier JC, Grosclaude F (1990). Two of the three genetic variants of goat alpha s1-casein which are synthesized at a reduced level have an internal deletion possibly due to altered RNA splicing. Eur J Biochem.

[12] Leroux C, Mazure N, Martin P (1992). Mutations away from splice site recognition sequences might cis-modulate alternative splicing of goat alpha s1-casein transcripts Structural organization of the relevant gene. J Biol Chem.

[13] Ptack E, Schaeffer LR (1993). Use of test day yields for genetic evaluation of dairy sires and cows. Livestock Prod Sci.

[14] Sanchez A, Ilahi H, Manfredi E, Serradilla JM (2005). Potential benefit from using the alpha(s1)-casein genotype information in a selection scheme for dairy goats. J Anim Breed Genet.

[15] Monti JC, Mermoud AF, Jolles P (1989). Anti-bovine beta-lactoglobulin antibodies react with a human lactoferrin fragment and bovine beta-lactoglobulin present in human milk. Experientia.

[16] Flower DR (1996). The lipocalin protein family: structure and function. Biochem J.

[17] Perez MD, Calvo M (1995). Interaction of beta-lactoglobulin with retinol and fatty acids and its role as a possible biological function for this protein: a review. J Dairy Sci.

[18] Folch JM, Coll A, Hayes HC, Sanchez A (1996). Characterization of a caprine beta-lactoglobulin pseudogene, identification and chromosomal localization by in situ hybridization in goat, sheep and cow. Gene.

[19] Folch JM, Coll A, Sanchez A (1994). Complete sequence of the caprine beta-lactoglobulin gene. J Dairy Sci.

[20] Kumar A, Rout PK, Roy R (2006). Polymorphism of beta-lactoglobulin gene in Indian goats and its effect on milk yield. J Appl Genet.

[21] Sardina MT, Rosa AJ, Davoli R, Braglia S, Portolano B (2012). Polymorphisms of beta-lactoglobulin promoter region in three Sicilian goat breeds. Mol Biol Rep.

[22] Arora R, Bhatia S, Mishra BP, Sharma R, Pandey AK, Prakash B (2010). Genetic polymorphism of the beta-lactoglobulin gene in native sheep from India. Biochem Genet.

[23] Mastrangelo S, Sardina MT, Riggio V, Portolano B (2012). Study of polymorphisms in the promoter region of ovine beta-lactoglobulin gene and phylogenetic analysis among the Valle del Belice breed and other sheep breeds considered as ancestors. Mol Biol Rep.

[24] Ganai NA, Bovenhuis H, van Arendonk JA, Visker MH (2009). Novel polymorphisms in the bovine beta-lactoglobulin gene and their effects on beta-lactoglobulin protein concentration in milk. Anim Genet.

[25] Vohra V, Kumar Bhattacharya T, Dayal S, Kumar P, Sharma A (2006). Genetic variants of beta-lactoglobulin gene and its association with milk composition traits in riverine buffalo. J Dairy Res.

[26] Morammazi S, Masoudi AA, Vaez Torshizi R, Pakdel A (2016). Changes in the Expression of the Prolactin Receptor (PRLR) Gene in Different Physiological Stages in the Mammary Gland of the Iranian Adani Goat. Reprod Dom Anim.

[27] Meyer K (2007). WOMBAT - Atool for mixed model analyses in quantitative genetics by REML. Journal of Zhejiang University-SCIENCE B.

[28] Pfaffl MW (2001). A new mathematical model for relative quantification in real-time RT-PCR. Nucleic Acids Res.

[29] Bustin SA (2002). Quantification of mRNA using real-time reverse transcription PCR (RT-PCR): trends and problems. J MolEndocrinol.

[30] Manjarin R, Steibel JP, Zamora V, Am-In N, Kirkwood RN, Ernst CW (2011). Transcript abundance of amino acid transporters, betacasein,and alpha-lactalbumin in mammary tissue of periparturient,lactating, and postweaned sows. J Dairy Sci.

[31] Bougarn S, Cunha P, Gilbert FB, Meurens F, Rainard P (2011). Technical note: Validation of candidate reference genes for normalization of quantitative PCR in bovine mammary epithelial cells responding to inflammatory stimuli. J Dairy Sci.

[32] Varshney N, Mohanty AK, Kumar S, Kaushik JK, Dang AK, Mukesh M (2012). Selection of suitable reference genes for quantitative gene expression studies in milk somatic cells of lactating cows (Bos indicus). J Dairy Sci.

[33] Vazquez-Flores F, Montaldo HH, Torres-Vazquez JA, Alonso-Morales RA, Gayosso-Vazquez A, Valencia-Posadas M (2012). Additive and dominance effects of the alpha(s1)-casein locus on milk yield and composition traits in dairy goats. J Dairy Res.

[34] Braunschweig MH, Leeb T (2006). Aberrant low expression level of bovine beta-lactoglobulin is associated with a C to Atransversion in the BLG promoter region. J Dairy Sci.

[35] Ollier S, Chauvet S, Martin P, Chilliard Y, Leroux C (2008). Goat's alphaS1-casein polymorphism affects gene expression profile of lactating mammary gland. Animal.

[36] Berget I, Martens H, Kohler A, Sjurseth SK, Afseth NK, Narum B (2010). Caprine CSN1S1 haplotype effect on gene expression and milk composition measured by Fourier transform infrared spectroscopy. J Dairy Sci.

[37] Caravaca F, Amills M, Jordana J, Angiolillo A, Aguera P, Aranda C (2008). Effect of alphas1-casein (CSN1S1) genotype on milk CSN1S1 content in Malaguena and Murciano-Granadina goats. J Dairy Res.

[38] Kuss AW, Gogol J, Bartenschlager H, Geldermann H (2005). Polymorphic AP-1 binding site in bovine CSN1S1 shows quantitative differences in protein binding associated with milk protein expression. J Dairy Sci.

[39] Graziano M, D’Andrea M, Angiolillo A, Lagonigro R, Pilla F (2003). A new polymorphism in goat b-lactoglobulin promoter region. Ital J Anim Sci.

[40] Ballester M, Sanchez A, Folch JM (2005). Polymorphisms in the goat betalactoglobulin gene. J Dairy Res.

[41] Chen H, Lan XY, Li RB, Lei CZ, Sun WB, Zhang RF (2005). The effect of CSN1 S2, CSN3 and beta-lg genes on milk performance in Xinong Saanen dairy goat. Yi Chuan Xue Bao.

[42] Prinzenberg EM, Weimann C, Brandt H, Bennewitz J, Kalm E, Schwerin M (2003). Polymorphism of the bovine CSN1S1 promoter: linkage mapping, intragenic haplotypes, and effects on milk production traits. J Dairy Sci.

[43] Lum LS, Dovc P, Medrano JF (1997). Polymorphisms of bovine beta-lactoglobulin promoter and differences in the binding affinity of activator protein-2 transcription factor. J Dairy Sci.

[44] Barbieri ME, Manfredi E, Elsen JM, Ricordeau G, Bouillon J, al al (1995). eEffects of the alpha(S1)-casein locus on dairy performances and genetic-parameters of alpine goats. Genet Sel Evol.

[45] Molenaar AJ, Wheeler TT, Grigor MR (2000). Nuclear localisation of the transcription factor Stat5b is associated with ovine milk protein gene expression during lactation but not during late pregnancy or forced weaning. Histochem J.

[46] Norgaard JV, Sorensen MT, Theil PK, Sehested J, Sejrsen K (2008). Effect of pregnancy and feeding level on cell turnover and expression of related genes in the mammary tissue of lactating dairy cows. Animal.

[47] Li HM, Wang CM, Li QZ, Gao XJ (2012). MiR-15a Decreases Bovine Mammary Epithelial Cell Viability and Lactation and Regulates Growth Hormone Receptor Expression. Molecules.

[48] Caroli AM, Chessa S, Erhardt GJ (2009). Invited review: milk protein polymorphisms in cattle: effect on animal breeding and human nutrition. J Dairy Sci.

[49] Chianese L, Portolano B, Troncone E, Pizzolongo F, Ferranti P,Addeo F, et al. The quality of Girgentana goat milk. Proceedings of the 9 International Conference on Goats; Tours, France 2000.p. 946-949.

